# Analysis of factors influencing onset and survival of patients with severe acute pancreatitis: A clinical study

**DOI:** 10.1002/iid3.1267

**Published:** 2024-06-18

**Authors:** Xiaoli Qin, Shili Xiang, Wenjing Li

**Affiliations:** ^1^ Gastroenterology Department The Third Affiliated Hospital of CQMU Chongqing China

**Keywords:** laboratory indicators, progression, severe acute pancreatitis, survival outcome

## Abstract

**Objectives:**

Acute pancreatitis (AP) is an inflammatory disease of the pancreas, and the prognosis of severe AP (SAP) is poor. The study aimed to identify promising biomarkers for predicting the occurrence and survival outcome of SAP patients.

**Materials and Methods:**

Two hundred and forty AP patients were retrospectively recruited, in which 72 cases with SAP. Blood test was done for collection of laboratory indicators. After treatment, the mortality of patients was recorded.

**Results:**

Patients in the SAP group had higher intensive care unit admissions and longer hospital stays (*p* < .001). Among laboratory parameters, significantly high values of C‐reactive protein (CRP), triglycerides and glucose (TyG) index, Von willebrand factor antigen (vWF:Ag) and D‐dimer were found in SAP groups relative to non‐SAP ones. Receiver operating characteristic curve indicated the good performance of CRP, TyG index, vWF:Ag and D‐dimer in SAP diagnosis. Among all SAP cases, 51 survived while 21 died. TyG index (odds ratio [OR] = 6.914, 95% confidence interval [CI] = 1.193–40.068, *p* = .028), vWF:Ag (OR = 7.441, 95% CI = 1.236–244.815, *p* = .028), and D‐dimer (OR = 7.987, 95% CI = 1.251–50.997, *p* = .028) were significantly related to survival outcome of SAP patients by multiple logistic regression analysis. Both TyG index and vWF showed favorable efficiency in predicting overall prognosis. The area under the curve for the multivariate model (PRE = −35.908 + 2.764 × TyG + 0.021 × vWF:Ag) was 0.909 which was greater than 0.9, indicating its excellent performance in prognosis prediction.

**Conclusion:**

CRP, TyG index, vWF:Ag, and D‐dimer values on admission may be potential clinical predictors of the development of SAP. Moreover, TyG index and vWF:Ag may be helpful to predict survival outcome.

## Graphical abstract

C‐reactive protein (CRP), triglycerides and glucose (TyG) index, Von willebrand factor antigen (vWF:Ag), and D‐dimer values on admission may be potential clinical predictors of the development of severe acute pancreatitis (SAP). Moreover, TyG index and vWF:Ag may be helpful to predict survival outcome.

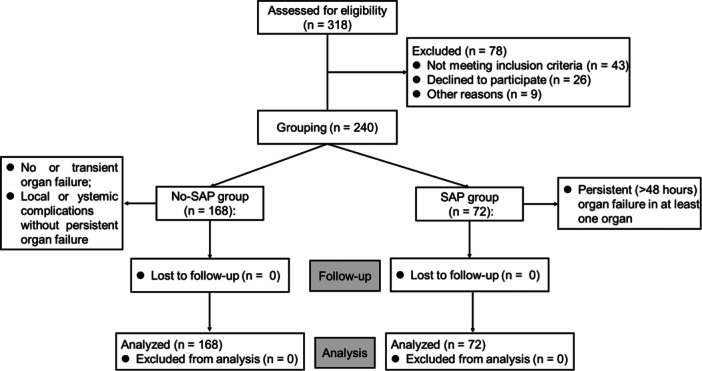

## 1 INTRODUCTION

Acute pancreatitis (AP) is a disorder characterized by parenchymal injury of the pancreas controlled by immune cell‐mediated inflammation.^1^ It is characterized by severe abdominal pain and elevated pancreatic enzymes with significant morbidity and mortality.^2^ The global incidence of AP is 34 people per 100,000 persons per year and it has been increasing worldwide.^3^ In recent years, the incidence of several obesity‐related complications has been on the rise, including cholelithiasis, hypertriglyceridemia, and diabetes, which are independently associated with AP.^4^ The exact pathophysiological mechanism of AP has been a mystery for decades, aside from the recognition that it may be a disease of the digestive system itself.^5^ Major cellular changes in the pathogenesis of AP include pathological calcium signaling, mitochondrial dysfunction, premature activation of trypsinogen in acinar cells and macrophages, endoplasmic reticulum stress, impaired unfolded protein response, and impaired autophagy.^6^ Immune cell‐derived inflammatory cytokines have been recognized to play critical roles in the pathogenesis of the disease. Despite the global burden of disease, there are currently no effective drugs to treat or prevent AP.^7^ The surviving patients of AP often develop long‐term dire outcomes, such as diabetes, exocrine pancreatic insufficiency, chronic pancreatitis, and reduced quality of life.^8^ Therefore, it is of great significance for guiding clinical treatment to find indicators that can predict the severity and poor prognosis of patients with AP.

The prognosis of mild AP (MAP) is generally good after short‐term treatment. However, severe acute pancreatitis (SAP) is often accompanied by organ dysfunction, systemic or local complications, and the prognosis is poor. Patients with SAP have variable and extremely rapid progression.^7^ If the treatment is not timely, SAP patients can be complicated by various diseases or infections, which greatly increases the risk of death.^9^ Therefore, early correct prediction of the severity and prognosis of SAP patients has guiding effects on the timely targeted intervention. At present, some laboratory indicators have been reported to reflect the severity of AP, such as blood urea nitrogen (BUN), neutrophil–lymphocyte ratio, creatinine, and so forth.^10^ In addition, lipid metabolism and inflammation are closely related to AP disease, and several related laboratory markers are determined to be related to AP diagnosis and prognosis, such as C‐reactive protein (CRP), white blood cells (WBC), and triglyceride (TG).^11^, ^12^ However, the study showed that the single index of AP disease assessment has limitations because of their dissatisfactory sensitivity or specificity.^13^

Recently, the triglycerides and glucose (TyG) index has been identified as a reliable surrogate marker for insulin resistance (IR) in numerous population‐based studies. It may predict the progress of various metabolic diseases, such as type 2 diabetes mellitus, and non‐alcoholic fatty liver disease (NAFLD).^14^, ^15^ Moreover, metabolic syndrome (MS), diabetes, dyslipidemia, and NAFLD have been reported to be risk factors for the occurrence of AP.^16^, ^17^ Therefore, it is reasonable to conjecture the relationship of TyG index with AP development. In addition, the inflammation caused by AP can lead to vascular endothelial damage, thus activating the coagulation system and disrupting the dynamic balance of the coagulation and anticoagulation systems.^18^ Therefore, the clinical value of coagulation index in AP has aroused our attention.

In the current study, 240 AP patients were retrospectively included in the present study, and their clinical laboratory indicators were recorded to explore their prognostic value in the development and clinical outcome of SAP patients. Specially, TyG index and coagulation index (Von willebrand factor antigen [vWF:Ag] and D‐dimer) were of significant concern.

## 2 MATERIALS AND METHODS

### 2.1 Study subjects

**Figure 1:**
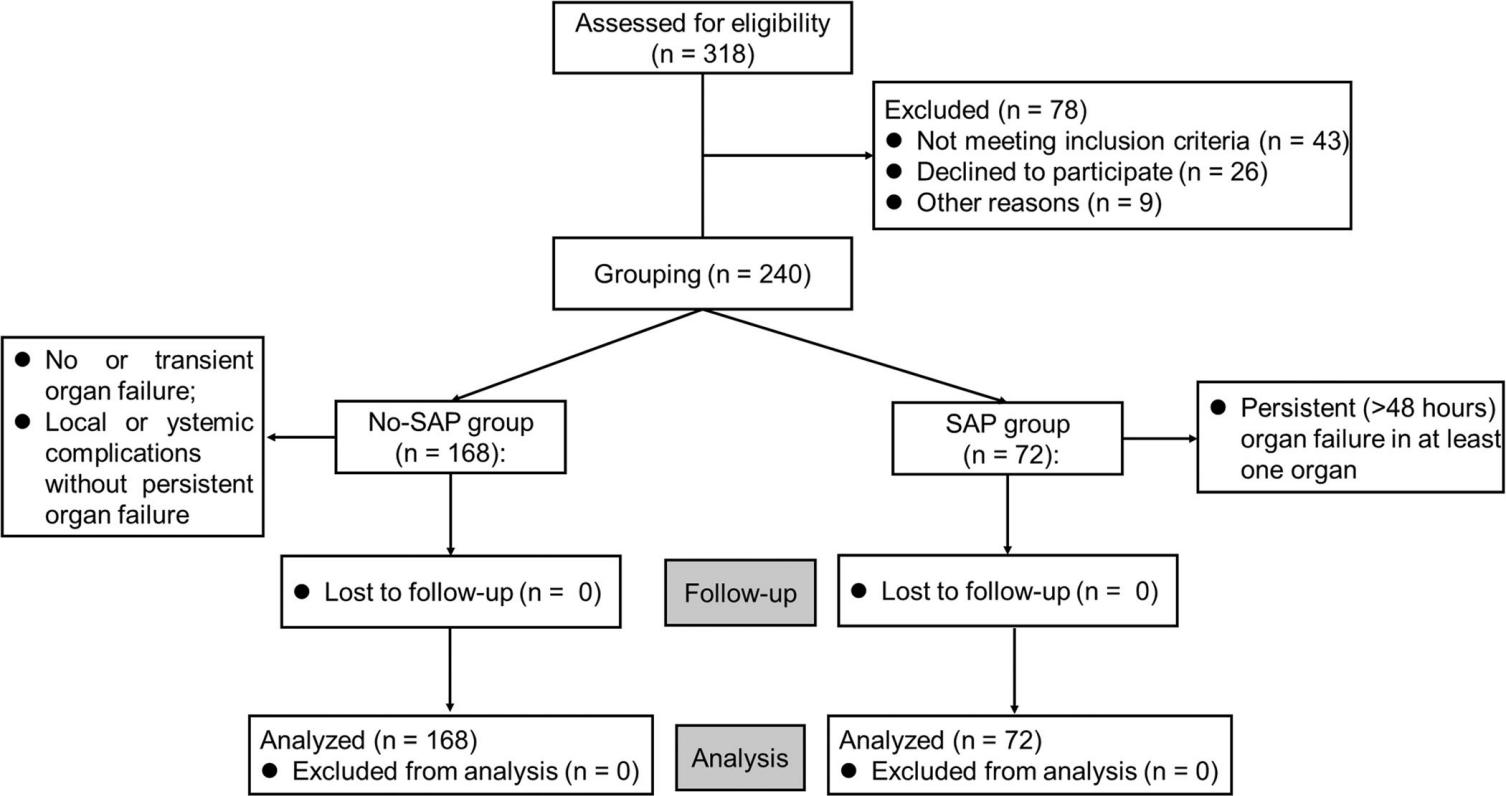
CONSORT flow diagram.

A total of 240 patients who were first diagnosed with AP and admitted to the Third Affiliated Hospital of CQMU from October 1, 2021, to July 1, 2023, within 48 h after onset were recruited in the present study, patient selection process was shown in Figure 1. The diagnostic criteria for AP were in line with the 2021 Chinese Guidelines for Diagnosis and Treatment of Acute Pancreatitis^19^: (1) persistent epigastric pain; (2) serum amylase and/or lipase concentrations three times higher than the normal upper limit; (3) abdominal imaging findings were consistent with AP. If two of the above three criteria are met, AP is diagnosed. Based on the 2012 Atlanta classification,^20^ the disease severity of AP was evaluated, all cases were divided into non‐SAP and SAP groups. Non‐SAP group includes cases with MAP and moderately severe AP (MSAP), while cases with SAP fell into the SAP group. MAP was defined as having no organ failure and local or systemic complications. MSAP is indicated by transient organ failure (<48 h) or local or systemic complications without persistent organ failure. Persistent (>48 h) organ failure in at least one organ is the characteristic of SAP. After treatment, the mortality of patients was recorded.

Exclusion criteria: (1) Patients with incomplete clinical data and incomplete blood tests within 24 h of admission. (2) Antibiotics and blood purification treatment have been applied before admission, and glucocorticoids, leukocyte‐enhancing preparations, anticoagulant drugs, and so forth, have been used before the onset of the disease. (3) Previously diagnosed patients with chronic pancreatitis, pancreatic tumor, posttraumatic and postoperative pancreatitis, and pregnancy with pancreatitis. (4) Patients with other acute infections, autoimmune diseases, malignant tumors, blood diseases, immune deficiency diseases, and chronic organ dysfunction.

### 2.2 Detection of laboratory indicators

Clinical data including age, gender, past medical history, and etiology were collected from each participant. Laboratory tests were accomplished within 24 h of admission. Levels of WBC, CRP, BUN, albumin, creatinine, calcium (Ca), fasting blood glucose (FBG), TG, uric acid were tested. The following formula was applied for the calculation of TyG index: TyG index = ln [TG (mg/dL) × FBG (mg/dL)/2]. Blood coagulation function indicators including platelets (PLT), vWF:Ag, and D‐dimer were recorded. All laboratory indicators were detected by an automatic blood cell analyzer (Sysmex XN9000) or automatic blood coagulation analyzer (Sysmex CS5100). Respiratory parameters were measured by arterial blood gas analysis, and the ratio of arterial oxygen partial pressure to fractional inspired oxygen (PaO_2_/FiO_2_) was calculated to assess the alveolar injury.

### 2.3 Statistical analysis

Based on three independent experiments results, the data were presented as mean and standard deviation (SD). Data analysis and figure visualization were performed using SPSS 21.0 and GraphPad statistical software. The statistical difference was analyzed using one‐way ANOVA followed by a Tukey's post hoc test or Student's test. The diagnostic significance was determined by plotting the receiver operating characteristic (ROC) curve. To identify key influence factors, univariate and multiple logistic regression analysis was carried out. *p* Value <.05 was set as the significant criteria.

## 3 RESULTS

### 3.1 Clinical data of the study population

According to inclusion and exclusion criteria, a total of 240 AP patients were enrolled in the present study, of which 72 cases with SAP (Table 1). There was no remarkable difference in age and gender distribution between SAP and non‐SAP groups (*p* > .05). The etiology and medical history of all cases were also recorded, and no significant difference was tested between the two groups (*p* > .05). It can be seen that patients in the SAP group had higher intensive care unit (ICU) admissions and longer hospital stays (*p* < .001). Among laboratory parameters, significantly high values of WBC, CRP, BUN, creatinine, Ca, PaO_2_/FiO_2_, FBG, TG, TyG index, vWF:Ag, and D‐dimer were found in SAP groups relative to non‐SAP ones, while significantly low values of albumin and PaO_2_/FiO_2_ were detected (*p* < .001). Although PLT values were lower in SAP cases than non‐SAP ones, the difference did not reach a significant level (*p* > .05).

**Table 1:** Basic characteristics and laboratory parameters of the study subjects.

Characteristics	Non‐SAP (*n* = 168)	SAP (*n* = 72)	*p* Value
Age (year)	49.23 ± 14.21	50.94 ± 12.98	.383
Sex (male/female)	99/69	43/29	.909
Etiology, *n* (%)			.290
Hypertriglyceridemia	34 (20.24)	18 (25.00)	
Gallstone	72 (42.86)	26 (36.11)	
Alcoholic	30 (17.86)	12 (16.67)	
Others	32 (19.05)	16 (22.22)	
ICU admission, *n* (%)	1 (0.60)	35 (48.61)	**<.001**
Hospital stays (days)	9.92 ± 4.51	19.11 ± 8.75	**<.001**
History, *n* (%)			
Hypertension	38 (22.62)	17 (23.61)	.867
Diabetes	41 (24.40)	22 (30.56)	.321
Smoking	50 (29.76)	21 (29.17)	.926
Drinking	72 (42.86)	32 (44.44)	.820
NAFLD	67 (39.89)	33 (45.83)	.391
Laboratory parameters			
WBC (×10^9^/L)	11.68 ± 3.84	13.24 ± 4.87	**.017**
CRP (mg/L)	63.66 ± 34.65	119.94 ± 27.67	**<.001**
BUN (mmol/L)	4.93 ± 1.90	6.58 ± 3.10	**<.001**
Albumin (mmol/L)	37.19 ± 5.21	34.64 ± 6.04	**.001**
Creatinine (mmol/L)	70.28 ± 18.78	125.46 ± 29.68	**<.001**
Ca (mmol/L)	2.10 ± 0.21	1.94 ± 0.31	**<.001**
PaO_2_/FiO_2_	338.09 ± 85.41	275.27 ± 81.29	**<.001**
FBG (mmol/L)	4.23 ± 1.74	5.49 ± 2.58	**<.001**
TG (mmol/L)	3.69 ± 1.98	5.97 ± 2.25	**<.001**
TyG index	9.18 ± 0.78	9.97 ± 0.66	**<.001**
Uric acid (μmol/L)	310.41 ± 107.06	332.13 ± 133.75	.225
PLT (10^9^/L)	173.08 ± 51.88	158.08 ± 62.16	.055
vWF:Ag (%)	220.47 ± 94.71	320.98 ± 79.91	**<.001**
D‐dimer (mg/L)	821.32 ± 204.70	2425.75 ± 1060.85	**<.001**

*Note*: Bold represents significant difference.

Abbreviations: BUN, blood urea nitrogen; CRP, C‐reactive protein; FBG, fasting blood glucose; ICU, intensive care unit; NAFLD, non‐alcoholic fatty liver disease; PaO_2_/FiO_2_, partial pressure of oxygen (PaO_2_)/fraction of inspired oxygen (FiO_2_); PLT, platelets; SAP, severe acute pancreatitis; TG, triglycerides; TyG, triglycerides and glucose; vWF:Ag: Von willebrand factor antigen; WBC, white blood cells.

### 3.2 Diagnostic value of laboratory indicators in predicting development of SAP for AP patients

**Figure 2:**
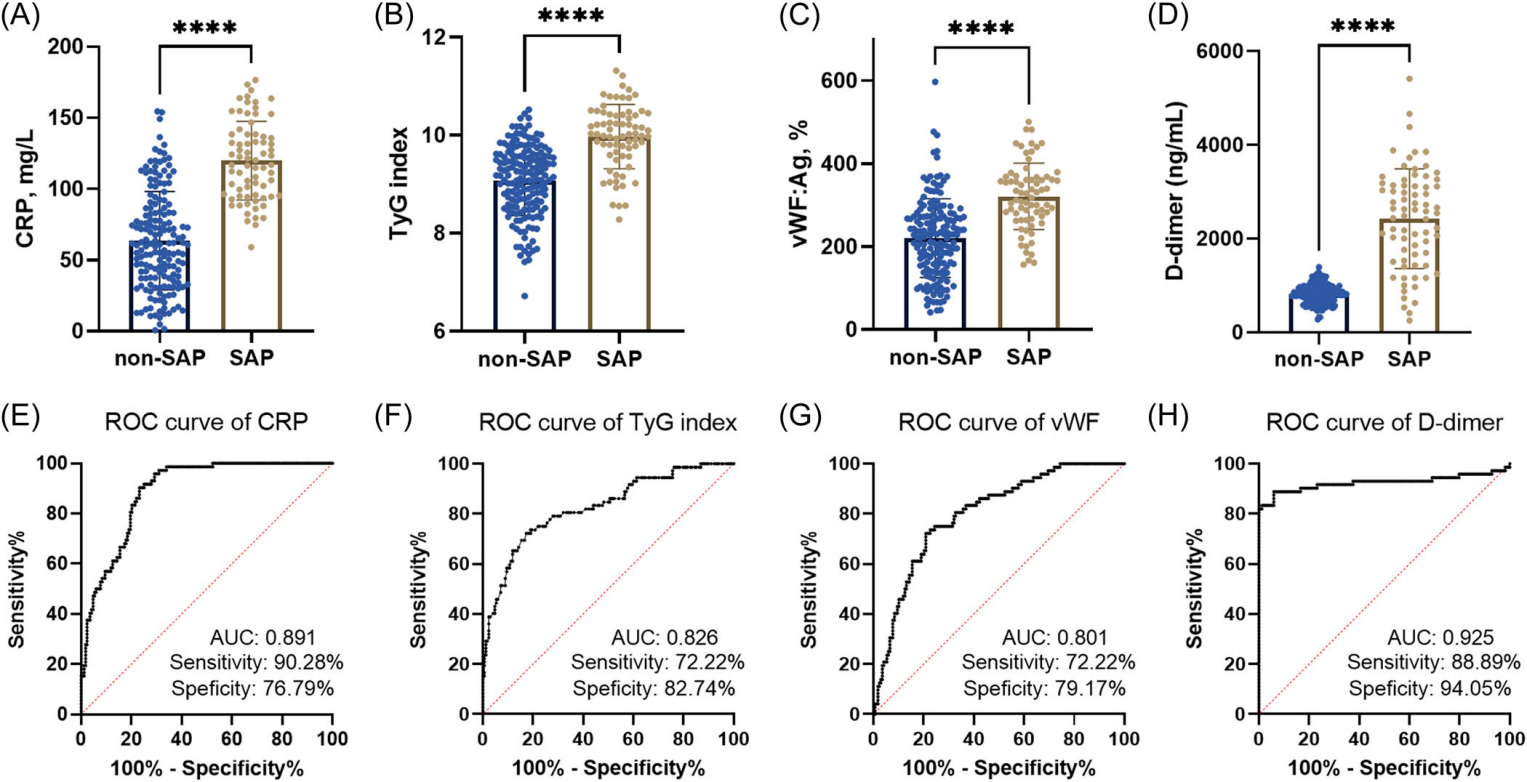
Diagnostic value of laboratory indicators in predicting development of severe acute pancreatitis (SAP) for AP patients. (A–D) Elevated levels of C‐reactive protein (CRP), triglycerides and glucose (TyG) index, Von willebrand factor antigen (vWF:Ag), and D‐dimer in SAP cases. (E–H) Receiver operating characteristic (ROC) curve of CRP, TyG index, vWF:Ag, and D‐dimer in SAP diagnosis. ****p < .0001.

As shown in Figure 2A–D, presented the elevated levels of CRP, TyG index, vWF:Ag, and D‐dimer in SAP cases. Moreover, their diagnostic significance was evaluated via plotting ROC curve. As shown in Figure 2E, the ROC curve indicated the high diagnostic value of CRP with the area under the curve (AUC) of 0.891. Then sensitivity and specificity were 90.28% and 76.79% respectively. Figure 2F presented the AUC (0.826) of TyG index in differentiating SAP from AP, the sensitivity and specificity were 72.22% and 82.74%, respectively. The high diagnostic value of vWF was determined in Figure 2G whose AUC was 0.801 with the sensitivity and specificity of 72.22% and 79.17%. The ROC curve indicated that D‐dimer had the best diagnostic value (AUC = 0.925) with a sensitivity of 88.89%, and a specificity of 94.05% (Figure 2H).

### 3.3 Clinical data analysis in survival group and non‐survival group

Based on the survival data, all SAP patients were classified into survival group (*n* = 51) and non‐survival group (*n* = 21). In comparison with survival group, the dead patients had high levels of CRP, BUN, creatinine, FBG, TG, TyG index, vWF:Ag, and D‐dimer on admission, while low level of albumin and PaO_2_/FiO_2_ (*p* < .001, Table 2). Moreover, high rate of ICU admission and long hospital stays were detected in non‐survival group (*p* < .05). Other index showed no significant difference between the two groups (*p* > .05).

**Table 2:** Basic characteristics and laboratory parameters of survival and non‐survival subjects.

Characteristics	Survival (*n* = 51)	Non‐survival (*n* = 21)	*p* Value
Age (year)	49.52 ± 12.18	54.40 ± 14.48	.148
Sex (male/female)	30/21	13/8	.809
Etiology			.980
Hypertriglyceridemia	13 (25.49)	5 (23.81)	
Gallstone	18 (35.29)	8 (30.95)	
Alcoholic	9 (17.65)	3 (14.29)	
Others	11 (21.57)	5 (23.81)	
ICU admission	19 (37.25)	16 (76.19)	**.003**
Hospital stays	17.49 ± 8.24	23.10 ± 8.99	**.013**
History			
Hypertension	11(21.56)	6 (28.57)	.525
Diabetes	14 (27.45)	8 (38.09)	.373
Smoking	15 (29.41)	6 (28.57)	.943
Drinking	23 (45.09)	9 (42.86)	.862
NAFLD	21 (41.18)	11 (52.38)	.384
Laboratory parameters			
WBC (×10^9^/L)	13.38 ± 4.57	12.91 ± 5.63	.713
CRP (mg/L)	115.06 ± 28.34	131.80 ± 22.38	**.018**
BUN (mmol/L)	5.67 ± 2.80	8.81 ± 2.66	**<.001**
Albumin (mmol/L)	36.81 ± 4.55	29.37 ± 6.06	**<.001**
Creatinine (mmol/L)	119.38 ± 28.85	140.25 ± 26.89	**<.001**
Ca (mmol/L)	1.98 ± 0.27	1.84 ± 0.38	.155
PaO_2_/FiO_2_	286.66 ± 83.46	247.62 ± 70.06	.064
FBG (mmol/L)	5.02 ± 2.21	6.62 ± 3.09	**.040**
TG (mmol/L)	5.62 ± 2.30	6.83 ± 1.91	**.037**
TyG index	9.76 ± 0.60	10.47 ± 0.50	**<.001**
Uric acid (μmol/L)	319.99 ± 119.47	361.59 ± 162.91	.233
PLT (10^9^/L)	156.90 ± 65.26	160.95 ± 55.29	.804
vWF:Ag (%)	294.23 ± 65.46	385.94 ± 75.50	**<.001**
D‐dimer (mg/L)	2243.87 ± 1142.60	2867.48 ± 664.88	**.005**

*Note*: Bold represents significant difference.

Abbreviations: BUN, blood urea nitrogen; CRP, C‐reactive protein; FBG, fasting blood glucose; NAFLD, non‐alcoholic fatty liver disease; ICU, intensive care unit; PaO_2_/FiO_2_, partial pressure of oxygen (PaO_2_)/fraction of inspired oxygen (FiO_2_); PLT, platelets; SAP, severe acute pancreatitis; TG, triglycerides; TyG, triglycerides and glucose; vWF:Ag: Von willebrand factor antigen; WBC, white blood cells.

### 3.4 Multiple logistic regression analysis of laboratory indicators in predicting survival outcome of SAP patients

**Figure 3:**
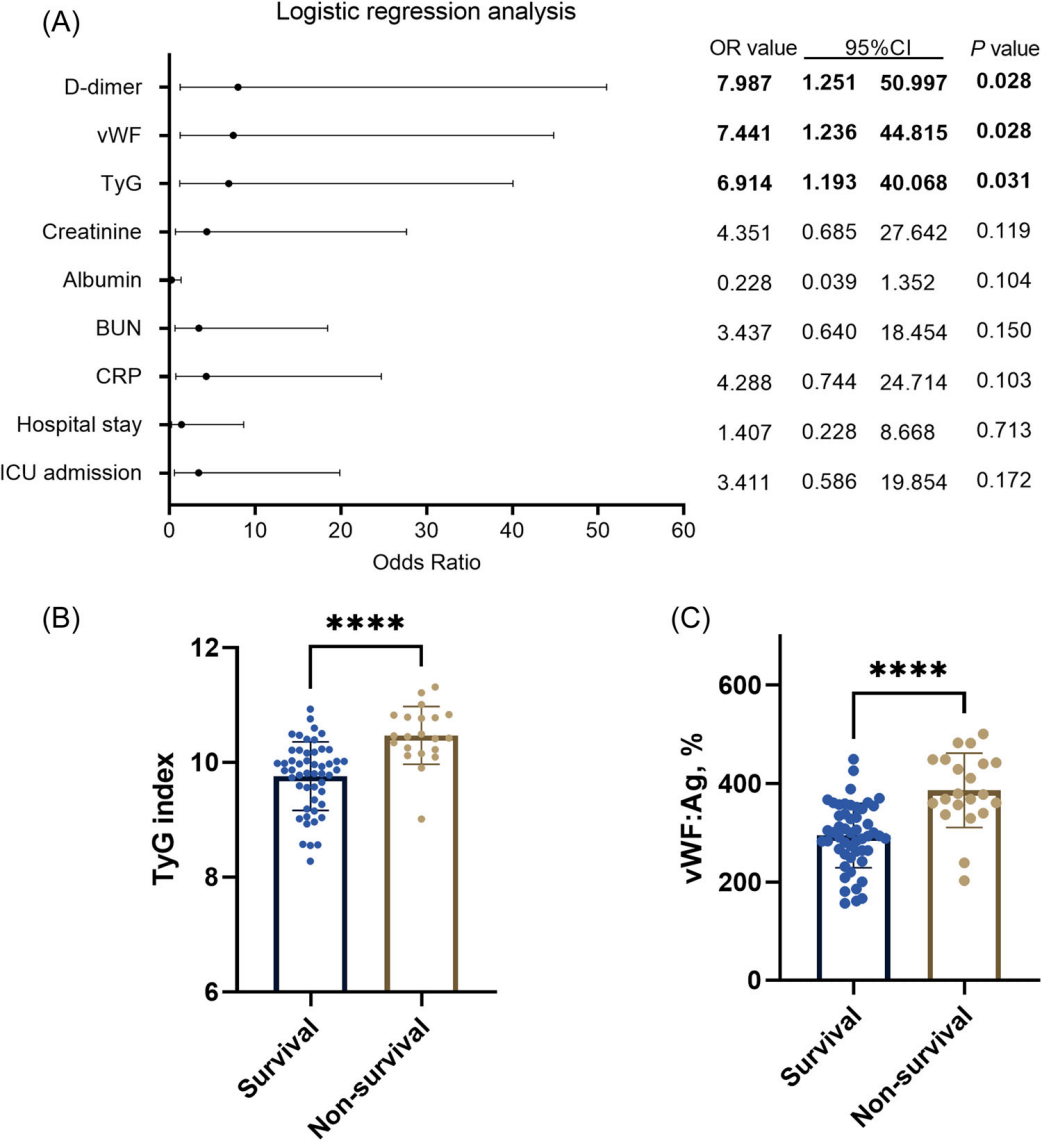
Multiple logistic regression analysis of laboratory indicators in predicting survival outcome of severe acute pancreatitis (SAP) patients. (A) Forest plot of multiple logistic regression analysis to show laboratory indicators related to survival outcome of SAP patients. (B and C) The high values of triglycerides and glucose (TyG) index and Von willebrand factor antigen (vWF:Ag) in non‐survival cases. ****p < .0001.

Then, we further identified indicators related to patients' survival. Based on the univariate analysis results, ICU admission, hospital stays, CRP, BUN, Albumin, creatinine, FBG, TG, TyG index, vWF:Ag, and D‐dimer were identified to be significantly related to patient's survival (Table 3, *p* < .05). Moreover, clinical parameters which showed significant differences were all introduced into multiple logistic regression model. After adjusting for other indicators, TyG index (odds ratio [OR] = 6.914, 95% confidence interval [CI] = 1.193–40.068, *p* = .028), vWF:Ag (OR = 7.441, 95% CI = 1.236–244.815, *p* = .028), and D‐dimer (OR = 7.987, 95% CI = 1.251–50.997, *p* = .028) were still significantly related to patients' survival (Figure 3A). Figure 3B,C presented the high values of TyG index and vWF:Ag in non‐survival cases (*p* < .001).

**Table 3:** Univariate analysis for prognostic factors in SAP patients.

Characteristics	OR value	95% CI	*p* Value
Age	1.828	0.647–5.163	.255
Sex	1.531	0.548–4.278	.416
ICU admission	3.667	1.253–10.730	**.018**
Hospital stays	3.215	1.111–9.309	**.031**
WBC	1.282	0.461–3.569	0.634
CRP	3.875	1.289–11.653	**.016**
BUN	3.524	1.220–10.179	**.020**
Albumin	3.667	1.253–10.730	**.018**
Creatinine	3.575	1.222–10.457	**.020**
Ca	1.989	0.702–5.631	.195
PaO_2_/FiO_2_	2.321	0.819–6.584	.113
FBG	2.200	0.781–6.197	.136
TG	2.667	0.941–7.560	**.065**
TyG index	5.602	1.651–19.015	**.006**
Uric acid	1.623	0.582–4.525	.354
PLT	1.562	0.553–4.411	.399
vWF:Ag	5.000	1.645–15.194	**.005**
D‐dimer	6.071	1.786–20.640	**.004**

*Note*: Bold represents significant difference.

Abbreviations: BUN, blood urea nitrogen; CRP, C‐reactive protein; FBG, fasting blood glucose; ICU, intensive care unit; PaO_2_/FiO_2_, partial pressure of oxygen (PaO_2_)/fraction of inspired oxygen (FiO_2_); PLT, platelets; SAP, severe acute pancreatitis; TG, triglycerides; TyG, triglycerides and glucose; vWF:Ag: Von willebrand factor antigen; WBC, white blood cells.

### 3.5 Prognostic value analysis of TyG index and vWF:Ag in predicting clinical survival of SAP patients

**Figure 4:**
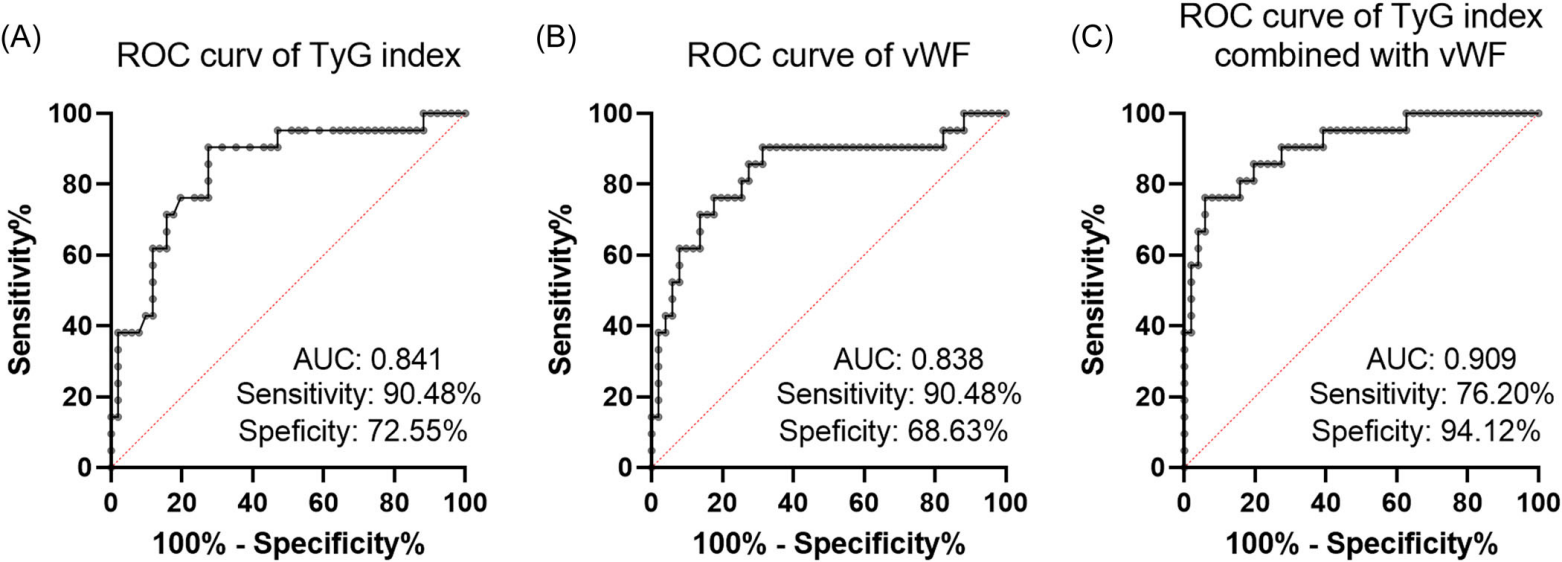
Prognostic value analysis of triglycerides and glucose (TyG) index and Von willebrand factor antigen (vWF:Ag) in predicting clinical survival of severe acute pancreatitis (SAP) patients. Receiver operating characteristic (ROC) of TyG index (A), vWF:Ag (B), and their combination (C) suggested their favorable efficiency in predicting overall prognosis.

The ROC diagnostic test was done to evaluate the diagnostic significance of TyG index and vWF:Ag in survival outcome. According to the ROC curve in Figure 4A, an AUC of 0.841 illustrated that the prognostic prediction value of TyG index for survival outcome of SAP patients was reliable, with the sensitivity of 90.48% and the specificity of 72.55%. The ROC of vWF:Ag for overall survival suggested a favorable efficiency of vWF in predicting overall prognosis (Figure 4B). Notably, the combination of TyG index and vWF:Ag showed superior performance in survival outcome of SAP patients. The AUC for the multivariate model (PRE = −35.908 + 2.764 × TyG + 0.021 × vWF:Ag) was 0.909 which was greater than 0.9, indicating its excellent performance in prognosis prediction (Figure 4C).

## 4 DISCUSSION

AP is characterized by local and systemic inflammatory responses and has a different clinical course.^21^ Most patients present with mild AP, which is self‐limiting and usually resolves within 1 week.^22^ About 20% of patients develop moderate or SAP with pancreatic necrosis, peripancreatic tissue, organ failure, or both, and the mortality rate is as high as 20%–40%.^23^ According to the present clinical data, approximately 30% AP cases developed into SAP among 240 cases. The progression of the disease was consistent with previous literature.^23^ For early detection and intervention of AP, the diagnostic values of clinical laboratory indicators were analyzed in AP patients. Moreover, the survival outcome of the SAP patients was also recorded, and TyG index and vWF:Ag were identified to be candidate prognostic biomarkers in predicting overall prognosis of SAP patients.

Early detection by SAP is essential for further management. A mass of studies have fully determined that MS, diabetes, dyslipidemia, NAFLD are risk factors for the onset and advance of AP.^24^ It is known that IR exerts an important influence on the development of chronic metabolic disorders.^25^ TyG index is a simple substitute index to evaluate IR, which has wide application in cardiovascular field.^26^ Based on the current results, elevated values of TyG index were detected in SAP cases compared with non‐severe ones, indicating its potential role in the advance of SAP. Moreover, its diagnostic value in SAP was further evaluated via plotting ROC curve. Based on the analysis results, it was determined that TyG index can differentiate SAP cases from non‐severe ones. TyG index was determined to be related to the severity of AP, and it was a promising reliable biomarker for predicting the advance of SAP. Consistently, in a latest study, TyG index is suggested to be an independent risk factor for SAP, moreover, its association with AP‐related complications was also determined.^27^ Moreover, according to our follow‐up results, a close relationship was also detected between TyG index and clinical prognosis of SAP patients. It was concluded that elevated TyG index can predict the advance of SAP, and is related to patients' poor prognosis.

CRP is a typical acute phase protein. It was originally found in the blood of patients infected with streptococcus pneumoniae.^28^ CRP concentrations began to rise 6 h after bacterial infection and reached a maximum after 24–48 h.^29^ It is reported that CRP is often elevated in AP patients and helps to assess the severity of the disease.^30^ Consistently, significantly elevated levels of CRP were detected SAP patients compared with non‐severe cases, indicating the diagnostic potential of CRP in SAP. Moreover, the ROC curve supported our conclusion. But as the follow‐up results indicated, elevated CRP was not independently related to the clinical prognosis of SAP patients.

Endothelial injury and coagulation dysfunction occur throughout the course of AP, which are also related to the disease severity.^31^ Previous studies have indicated that some hemostatic system‐related parameters are closely related to the severity and clinical prognosis of AP.^32^ In the present study, the SAP cases owned significantly elevated values of both D‐dimer and vWF:Ag relative to non‐severe cases. D‐dimer is a specific degradation product of crosslinked fibrin, which reflects the activity of thrombin and plasminase in vivo.^33^ It has been noted in the literature that D‐dimer is abnormally expressed in AP patients and is associated with disease severity, the evidence supported our findings in the current study.^34^ vWF:Ag is a macromolecular glycoprotein synthesized by vascular endothelial cells, which is one of the sensitive indexes to reflect whether blood vessels are damaged. When microvessels are damaged, vwF:Ag is released in large quantities.^35^ Elevated vWF:Ag in SAP patients indicated their early vascular endothelial injury in the present study. Besides, according to the survival data, cases in the non‐survival group showed higher values of vWF:Ag than those in the survival group. The findings indicated that early vascular endothelial injury was related to the poor prognosis of SAP patients, and vWF:Ag serves as a promising biomarker for protecting the clinical outcome of SAP patients. Notably, the combination of TyG index and vWF:Ag showed superior performance in survival outcome of SAP patients. The AUC for the multivariate model was greater than 0.9, indicating its excellent performance in prognosis prediction.

In conclusion, CRP, TyG index, vWF:Ag, and D‐dimer values on admission may be potential clinical predictors of the development of SAP. Moreover, TyG index and vWF:Ag may be helpful to predict survival outcome, and their combination showed excellent performance in prognosis prediction.

## AUTHOR CONTRIBUTIONS

**Xiaoli Qin**: Conceptualization; writing—original draft. **Shili Xiang**: Conceptualization; writing—original draft. **Wenjing Li**: writing—review & editing.

## CONFLICT OF INTEREST STATEMENT

The authors declare no conflict of interest.

## ETHICS STATEMENT

All procedures performed in studies involving human participants were in accordance with the ethical standards of the institutional and/or national research committee and with the 1964 Helsinki Declaration and its later amendments or comparable ethical standards. The study was approved by the Bioethics Committee of The Third Affiliated Hospital of CQMU (Approval Number: CQMU202011023R). Written informed consent was obtained from all subjects involved in the study.

## Data Availability

The data sets used and/or analyzed during the current study are available from the corresponding author on reasonable request.
